# Differences in central symptoms of anxiety and depression between college students with different academic performance: A network analysis

**DOI:** 10.3389/fpsyg.2023.1071936

**Published:** 2023-02-28

**Authors:** Yu Wang, Shuo Zhang, Xiaogang Liu, Hongye Shi, Xuyang Deng

**Affiliations:** ^1^Centre of Mental Health Education, Southeast University, Nanjing, Jiangsu, China; ^2^Centre of Mental Health Education, Yangzhou University, Yangzhou, Jiangsu, China; ^3^School of Humanities, Southeast University, Nanjing, Jiangsu, China; ^4^School of Mechanical Engineering, Southeast University, Nanjing, Jiangsu, China

**Keywords:** mental health, college students, depression, anxiety, academic performance, network analysis

## Abstract

**Objective:**

The prevalence of anxiety and depressive symptoms for Chinese college students are high. Academic pressure is one of the prominent risk factors of psychological well-beings for Chinese college students. The application of network analysis provides researchers a more comprehensive understanding of symptom-symptom interaction in mental disorders. This study aims to find out whether there is a difference in central symptoms between students with different academic performance.

**Method:**

A total sample of 1,291 college students was included in our study. Anxiety and depressive symptoms were measured by PHQ-9 and GAD-7. Central symptoms were identified through centrality indices. Network stability was examined using the case-dropping method.

**Results:**

For the poor academic group, the most central symptom is PHQ-2 (feeling depressed). The most central symptom of the good academic group is GAD-2 (uncontrolled worry). The least central symptom for both groups is PHQ-9 (suicidal thought). Network structure is statistically different between two groups, global strength is not statistically different between two groups.

**Conclusion:**

The pertinent symptom is feeling depressed, followed by uncontrolled worry and poor appetite, and for the good academic group, the pertinent symptom is an uncontrolled worry, theoretical explanation and clinical implications is discussed.

## 1. Introduction

Academic pressure is linked to mental health problems. For example, previous research has identified that academic stress is a risk factor for depression symptoms (Jayanthi et al., 2015). Fear of poor grades, exams, and assessments, lack of subject proficiency, and guilt from making mistakes in assignments are all risk factors associated with anxiety, depression and stress (Mofatteh, 2021). Academic performance is highly associated with depression (Turner et al., 2012; Gao et al., 2020). Academic stress is found to be linked to suicidal ideation, partially triggered by depression (Awadalla et al., 2020). Parent’s expectations, personal values, and school support impact both major depressive symptoms and academic performance. Although, parents’ expectations in certain cases have a positive relationship with academic performance; parents’ expectations have also been positively correlated with depression (DeRoma et al., 2009; Ma et al., 2018).

Researchers have shown that the prevalence of depressive symptoms among college students in China is 23.8%, and the prevalence of anxiety is 7.3% under the impact of COVID-19 (Guan et al., 2021; Lei et al., 2021). In the survey conducted in 2020 which compared data collected from 2014, the mental health issues in Chinese college students were targeted. Chinese graduates showed greater scores in all 9 dimensions in Symptom Checklist-90, with controlled variables, and academic stress is one of the significant predictors of higher SCL-90 scores (Lei et al., 2021).

Academic stress is influenced by both social and cultural contexts in China. Challenges faced by children may come from social or school environments. A multitude of parents have high expectations and shape their children, for university preparation from a tender age. Competitive examinations in school settings are a source of strain for children, consecutive examinations put enormous stress upon children (Zhao et al., 2015). Recently, it is observed that entering college is not the end of academic pressure. According to the ministry of education of China, in 2022; 4.57 million applicants are taking the graduate entrance exams, which is 0.8 million more than in 2021 (Xu and Liu, 2022). China’s higher education has been transformed from elite education to mass higher education within a very short span, meaning that the gross enrollment rate for colleges is over 15% and below 50% (Xiong et al., 2022). With the expansion of higher education, lots of obstacles arise, one specific problem is “credential inflation.” Credential inflation is defined as the devaluation of educational credentials in a job market, while the requirement of credentials for jobs keeps multiplying (Collins, 2002). On account of the credential inflation, a large number of students choose to apply for graduation to be competitive in the job markets. There is a specific program in several Chinese universities called “bao yan,” which refers to graduate school recommendation programs, students with better grades, could skip the graduate entrance exam and give interviews directly, therefore, academic achievement also poses competition in college settings, in China.

In recent times, the application of a new approach of statistical analysis in psychopathology called ‘network analysis’ which conceptualizes disorders as systematically connected symptoms rather than as latent disorders has been introduced (Borsboom and Cramer, 2013). Unlike traditional reflective latent approaches, which based on the assumption that co-occurring symptoms have underlying common causes, each individual symptom of mental health disorder in network analysis can be viewed as a “node,” which are connected by “edges” (Schmittmann et al., 2013). Mental health disorders are viewed as interaction of symptoms in network analysis, and the change of individual symptom or “node” can influence other symptoms in a network structure. Network analysis as a symptom-oriented approach can be used to identify the central symptoms, which represents the symptom with the strongest connections with other symptoms. Symptoms which are central to the network can be targeted as the prominent symptom to focus on in the clinical practice and influence the treatment of other symptoms. The application of network analysis has previously been implemented in different groups, for example, Mullarkey et al. (2018) have identified that self-hatred is the most central symptom in adolescents, since forming a positive identity is the most critical conflict during the stage of adolescence.

In terms of Chinese samples, Bai et al. (2021) used network analysis to identify the core symptoms and bridge symptoms of anxiety and depression in nursing students and found that irritability and anxiety were two primary core symptoms of nursing students, during the COVID-19 pandemic. Network analysis can also be applied to the comparison of central and bridge symptoms in two varied groups or two different periods. Wang et al. (2020) demonstrated that psychomotor symptoms impaired motor skills, restlessness, and inability are central symptoms observed during the period of COVID-19 outbreak, and its concentration decreased after the peak stage. Further, the comparison of network structure can be evaluated through network analysis, its application is widely used in cross-cultural studies, for instance, the psychopathy network for U.S prisoners showed similarities between U.S students and Chinese prisoners (Wang et al., 2022).

The COVID-19 raised attention of mental disorders in college students global widely. Depression and anxiety are two most common diseases in college students. Furthermore, depression and anxiety are co-occurring at high rates, the co-occurrences of anxiety and depression are explained in many studies (e.g., Gorman, 1996; Belzer and Schneier, 2004; Bitsika and Sharpley, 2012). Under the network analysis, psychopathology is based on the causal systems perspective (Borsboom, 2008), which the comorbidity is due to direct interaction between symptoms. The symptoms of a mental disorder can lead to the development of another mental disorder, to be specific, symptoms of depression can lead to the development of anxiety symptoms. Many previous studies focus on the symptom-symptom interaction of anxiety and depression psychopathology networks (e.g., Cramer et al., 2010; Wang et al., 2020; Bai et al., 2021).

Due to the overall education background, the stressors faced by students with good and poor academic performance were different. Thus, identifying the core symptoms of most common psychological disorders in different student groups based on their academic performance could provide insights for clinical practitioners. The current study aims to utilize the network analysis approach to (1) characterize the symptom-symptom interaction in depression and anxiety psychopathology network in students with different academic performance; (2) identify the core symptoms in different academic performance groups; (3) comparing the network differences in two groups.

## 2. Method

### 2.1. Participants

Participants were recruited from three universities in Jiangsu province of China, stratified cluster sampling method was adopted to collect data in different grades and majors. Freshmen, sophomore, and junior year students were included in our study, and majors included; science and engineering, arts and literature, and medicine. Data was collected on June 2021, which was not during the peak stage of COVID-19. The survey was conducted by a trained graduate student, questionnaires were administered in the classroom, and formal consent and instructions were provided before the survey. All students participated in this study voluntarily, no class credit or monetary awards were given. Collectively, 2,371 questionnaires were included in this study, 1,262 (53.20%) participants were male, and 1,109 (46.80%) participants were female. 666 (28.10%) participants were freshmen, 1,094 (46.10%) participants were sophomore, and 611 (25.80%) participants were junior, 816 (34.4%) participants’ grades ranged top 27% in their cohort, 1,080 (45.6%) ranged middle, and 475 (20%) ranged bottom 27%. This study was approved by the Research Ethics Committee at Yangzhou University.

### 2.2. Measurement

#### 2.2.1. Demographic variables

Basic demographic information was collected, including gender, major, current residence (rural or urban), and status of children in a family, were collected. As different majors may have a large difference in the mean grade point average (GPA), this study used grade percentile (top 27%, middle, bottom 27%) to capture the academic performance.

#### 2.2.2. Depression

Depression was surveyed by a 9-item patient health questionnaire-9 (PHQ-9). The PHQ-9 proved to be valid and reliable in varied languages by previous scholars (Kroenke et al., 2001; Wang et al., 2014). PHQ-9 is a four-point Likert scale, it ranges from 0 to 3, indicating “not at all,” “several days,” “more than half the days,” and “nearly every day” respectively, total scores ranged from 0 to 27. The cutoff score for the Chinese version of PHQ-9 is 5, scores over 5 represent individuals who have depressive symptoms. The Cronbach alpha for students with good and poor academic performance is 0.859 and 0.875, respectively.

*Anxiety*: Anxiety was measured by a 7-item generalized anxiety disorder scale (GAD-7). The scale is a four-point Likert scale, with each item ranging from 0 to 3, a total score ranging from 0 to 21, and higher scores indicating more severe symptoms of anxiety (Spitzer et al., 2006). The cutoff score for GAD-7 is also 5, scores over 5 indicate mild to high anxiety. The Cronbach alpha for students with good and poor academic performance is 0.915 and 0.928, respectively.

### 2.3. Statistical analysis

Firstly, a preliminary analysis was conducted by SPSS v.25. Two sample *t*-tests were conducted to compare means of anxiety and depression scores in different student groups. A *p*-value of 0.05 indicates the significance of the test.

All network analysis is conducted through R, and it is used for network analysis of mgm, qgraph, bootnet, networktools, and Network Comparison Test. In network structure, each item or symptom is called “nodes,” and the relationship between nodes is called “edges.” Based on visual representation, the thicker the edge, the stronger the magnitude of the correlation. Green and red color edges represent the positive and negative associations. In our study, edges were computed by the Extended Bayesian Information Criterion (EBIC) method. The graphic lasso (glasso) model was applied, to demonstrate a sparse representation of the network by shirking all edges in the network, and to reduce the possibility of false positive edges (Epskamp et al., 2012). In order to capture the variance that explained by other nodes, predictability of each node was measured (Haslbeck and Waldorp, 2015).

To capture the centrality of symptoms, indices of strength, closeness, and betweenness were calculated. Strength refers to the sum of weights that directly connect the nodes; closeness represents the inverse of the sum of all short pathways connecting other nodes; betweenness is calculated by the frequency of the shortest path that passes through two other nodes (Opsahl et al., 2010). The accuracy of the robustness of the network structure (edge weights) was evaluated by the non-parametric bootstrapping method. The accuracy was estimated by estimating the bootstrap confidence interval around the edges of 1,000 bootstrap samples from the data. And the centrality stability was assessed by the case-dropping bootstrapping resampling method (Epskamp et al., 2017), by exploring how the centrality indices changed when the proportion of cases dropped. The rapid change of significance indicated the lower stability of the centrality. As per previous researchers, the minimum case dropped should be over 25% and recommended must be over 50%, and the correlation of centrality indices for original networks and centrality indices for case-subset networks should remain at least at 0.7 (default setting of *boot net* package).

The Network Comparison Test package compared the two networks. Three invariant measures can be assessed through NCT: (1) invariant network structure, comparing whether the overall network structure of two populations is identical or not; (2) invariant edge strength, comparing the distribution of edge strength of the networks for two populations; (3) invariant global strength, comparing the overall connectivity of networks (Van Borkulo et al., 2022). The significance level was set to 0.05 to identify that the two networks were statistically different.

## 3. Results

As represented in Table 1, the mean depression scores; for students with poor academic performance were 9.81, with a standard deviation of 5.70; and for students with good academic performance was 6.29, with a standard deviation of 4.51. The poor academic group had mean anxiety scores of 8.09, with a standard deviation of 5.14; in the good academic group mean anxiety score was 6.01, with a standard deviation of 4.79. The mean scores of anxiety and depressive symptoms for poor academic students are significantly higher than for good academic students (*t* = −11.522, *p* < 0.01; *t* = −7.315, *p* < 0.001).

**Table 1:** Descriptive statistics and one-way ANOVA of anxiety and depression across two groups.

Academic performance	*N*	Depression Mean	Depression SD	Anxiety Mean	Anxiety SD
Bottom 27%	475	9.81	5.70	8.09	5.14
Top 27%	816	6.29	4.51	6.01	4.79
		*t* = −11.522; *p* < 0.001		*t* = −7.315; *p* < 0.001	

**Figure 1:**
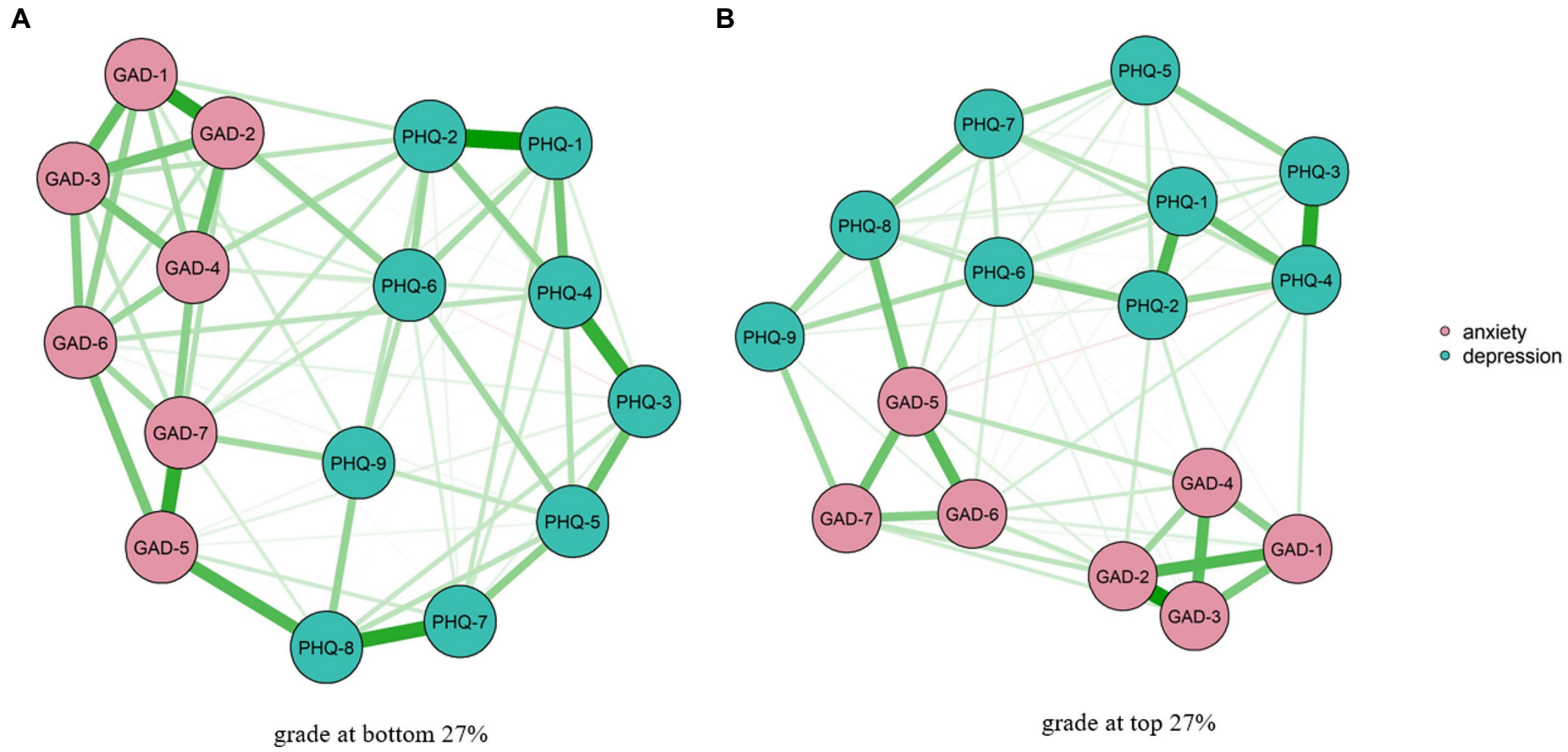
Network structure of anxiety and depressive symptoms among two groups.

As shown in Figure 1, students’ grades at the bottom 27% (poor academic group) of 16 nodes demonstrated that 73 out of 120 edges were non-zero, and the edge of GAD-1 (nervousness) and GAD-2 (uncontrolled worry) showed the strongest connection in the anxiety symptoms. PHQ-1 (low interest) and PHQ-2 (feeling depressed) demonstrated the strongest connection in the poor academic group of depressive symptoms. Students with grades in the top 27% (good academic group) of 16 nodes showed that 77 out of 120 edges were non-zero. GAD-2 (uncontrolled worry) and GAD-3 (excessive worry) showed the strongest connection among all edges in the anxiety symptoms, and PHQ-3 (trouble sleeping) and PHQ-4 (tired or little energy) had the strongest connection among all edges in the depressive symptoms. Non-parametric bootstrapped confidence intervals for edge weights and correlation matrices are provided in Supplementary Figure S1 and Supplementary Tables S1, S2. The mean predictability of good and poor academic group was 58.60 and 60.11%, demonstrating that on average 58.6 and 60.11% of each node’s variance explained by neighboring nodes (Supplementary Tables S3, S4).

**Figure 2:**
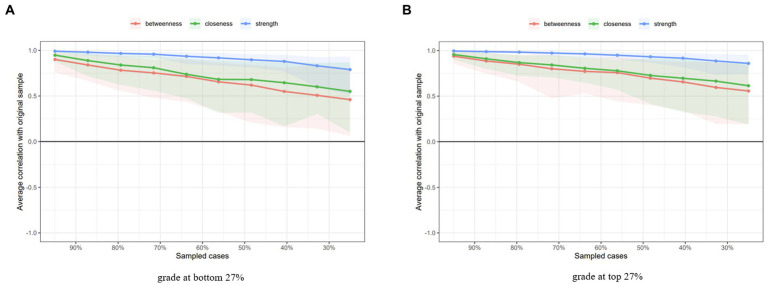
The stability of network structure by case dropping subset bootstrap.

The network accuracy and stability of centrality indices displayed in Figure 2 results, of the case dropping method, showed that strength remained stable for both samples, while betweenness and closeness showed poor stability. CS-Cs for strength, closeness, and betweenness were 0.594, 0.206, and 0.051, respectively, for the poor academic group; and 0.750, 0.361, and 0.206 for the good academic group. This indicates that, for both groups, the index of strength dropping 59.4 and 75.0% had no significant impact on the results, and remained over a 0.7 correlation coefficient. Non-parametric 95% C for edge weights, for both samples, were narrow and the edge values were statistically higher than zero, representing that these edge weights were stable (Supplementary Figure S1). Bootstrap difference test for edge weights and node strength provided in Supplementary Figure S2.

The stability of closeness and betweenness for both poor academic samples and the good academic sample is lower than 0.5, which does not reach the preferred level of stability, so the centrality of symptoms solely focused on strength. For the poor academic group, the central symptom is PHQ-2 (feeling depressed), followed by GAD-2 (uncontrolled worry) and GAD-5 (poor appetite). The least central symptom for both groups is PHQ-9 (suicidal thought). The central symptom of the good academic group is GAD-2 (uncontrolled worry), followed by PHQ-4 (tired or little energy) and GAD-3 (excessive worry).

The network comparison test demonstrated poor academic groups and the good academic group had a significant difference in network structure (*M* = 0.211, *p* = 0.034), indicating the distribution of edge weights that were not equal. However, the global edge strength for the two groups was not statistically different (poor academic group = 7.393, good academic group = 7.247, S = 0.147, *p* = 0.280). In terms of edge strength invariance; edge weights between PHQ-2 (feeling depressed) and GAD-7 (feeling afraid), GAD-3 (excessive worry) and GAD-6 (irritability), PHQ-9 (suicidal thoughts) and GAD-1 (nervousness) were larger in the poor academic group (*p* = 0.036; *p* = 0.019; *p* = 0.010), whereas, edge weights between PHQ-3 (trouble sleeping) and PHQ-6 (guilt), GAD-2 (uncontrolled worry) and GAD-3 (excessive worry) were larger in the good academic group (*p* = 0.001; *p* = 0.003), respectively.

## 4. Discussion

**Figure 3:**
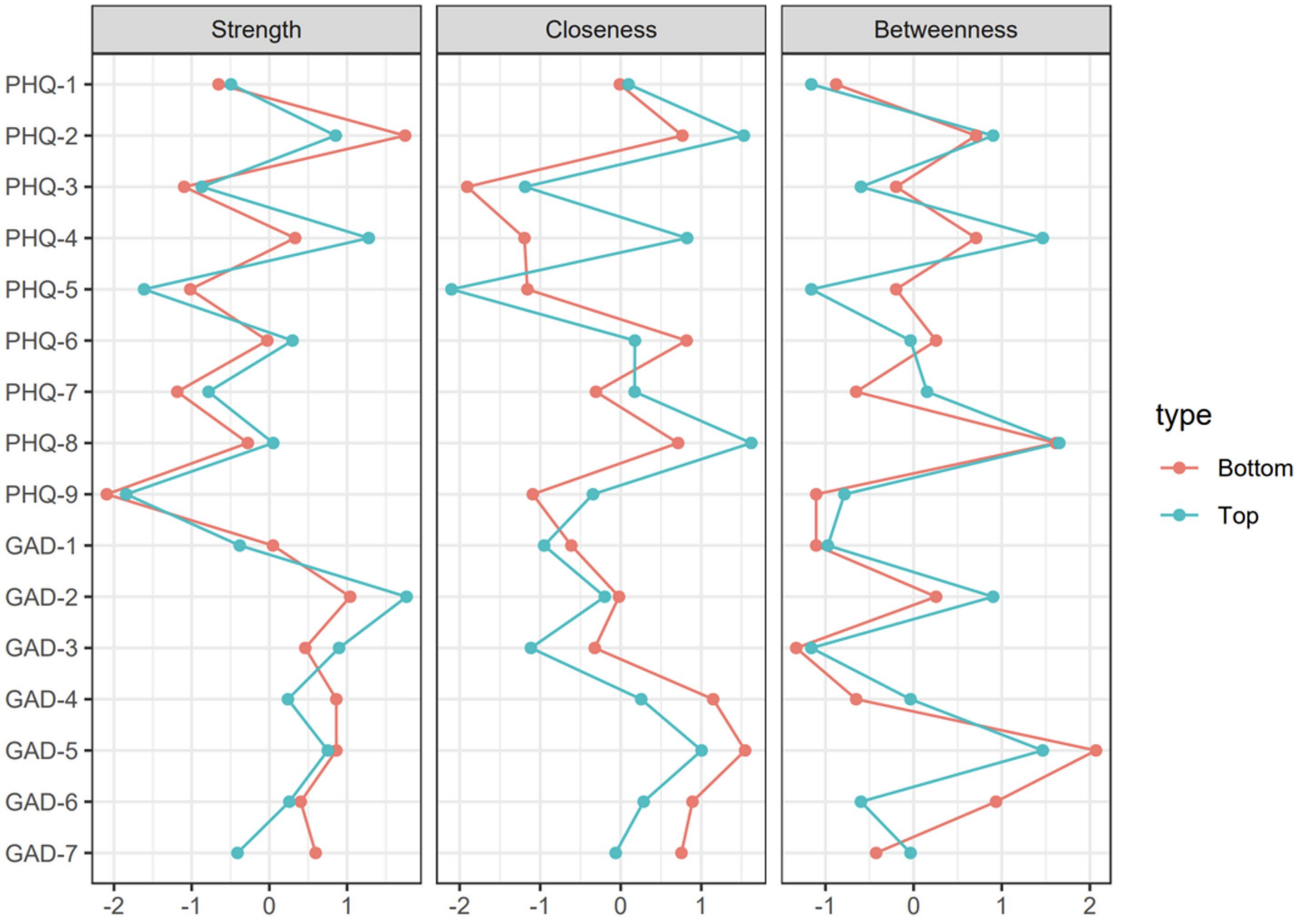
Standardized centrality indices of network structure of anxiety and depressive symptoms (z-scores).

The application of network analysis to psychological symptoms of anxiety and depression has been used in different populations, however, the majority of comparisons of networks focused on demographic variables, such as gender differences and residential differences (Bai et al., 2021; Zhao et al., 2021; Liu et al., 2022) or retrospective/prospective studies (Beard et al., 2016; Liu et al., 2022). The present study is the first to investigate the network structure, stability of edge weights and centrality indices, and prominent symptoms of college students with different academic performances. In general, the network structure is statistically proven to be significantly different in two groups, the central symptoms for the poor academic group is sad mood, followed by uncontrolled worry and poor appetite; and for the good academic group, it is uncontrolled worry, followed by tired or little energy and excessive worry (Figure 3).

The average depression and anxiety scores for students with poor academic performance were 9.81 and 8.09, and for students with good academic performance were 6.29 and 6.01. Our samples demonstrated relatively higher depressive and anxiety symptoms, compared to previous studies, and the majority of students showed some or the other symptoms of depression and anxiety, this could be due to data collection being in June, which usually was the final exam week. The anxiety and stress levels are particularly high during the examination period for college students (Gallego et al., 2014). Therefore, academic performance is related to psychological well-being, to be specific, students with poor academic performance were associated with higher anxiety and depression levels, in correspondence to previous research (Eisenberg et al., 2009; Dong et al., 2021).

Feeling depressed or sad is a critical symptom in the poor academic sample, the role of depressed mood as being a prominent symptom has been proved by many scholars, and our study is consistent with their findings (Beard et al., 2016; Fried et al., 2016; Liu et al., 2022). There is a correlation between academic performance and self-efficacy, researchers proposed that intervention of enhancing performance could be done by raising self-efficacy (Lane et al., 2004). Self-efficacy, proposed by Albert Bandura, refers to one’s belief that one can execute behaviors necessary to reach a specific performance (Bandura, 1977). The influence of self-efficacy covered various psychological disciplines, including smoking cessation, work-related behaviors, sports skill performance, and academic performance (Honicke and Broadbent, 2016). In the academic field, self-efficacy usually is called academic self-efficacy (ASE), ASE has been studied in different periods, including early years, high school tears, and university levels (Honicke and Broadbent, 2016). Robbins et al. (2004) identified that the best predictors of grade average points (GPA) are ASE and achievement motivation. Self-efficacy influences depression in three ways: First, individuals feel unable to reach ideal performance that would bring satisfaction; Second, individuals with low self-efficacy believe that they cannot build a supportive relationship with others; Third, they believe that they are unable to control disturbing depressive ruminations (Maddux and Meier, 1995). Self-efficacy has both direct and indirect effects on preventing depression (Holahan and Holahan, 1987). Another research points out that self-efficacy mediates emotional regulation and academic performance (Usán Supervía and Quílez Robres, 2021). The previous research regarding self-efficacy could help understand why the depressed mood is the central symptom in students with lower grades.

Students with good academic performance, on the contrary, have their central symptoms as general anxiety disorder, and uncontrolled worry. Uncontrolled worry has been identified as a chief symptom in various populations, but not as the component symptom, of anxiety, the main symptoms of anxiety are irritability for nursing students or excessive worry for sub-Saharan African students (Osborn et al., 2020; Bai et al., 2021). The definition of worry is characterized as a process rather than a state of being, worry involves a process whereby the individual is preoccupied with a potential threat (Kelly and Miller, 1999). Kelly and Miller (1999) also mentioned three primary beliefs that worriers contain: fear, ‘must’ and ‘should’, and feelings of inadequacy. Fear of a loss of another person or loss of self-esteem; can identify suitable or concrete solutions to resolve personal problems or dilemmas.

Anxious individuals usually have high expectations and strive to be perfectionists. Meta-analysis has shown that there is a positive relationship between perfectionistic strivings and academic achievements (Madigan, 2019). While comparing students from western culture, Chinese students’ academic motivational goals to compete with others to get good grades and to be rewarded for their performance significantly correlated with anxiety, whereas western students do not show such correlation (Essau et al., 2008). Therefore, students with good academic performance usually have higher expectations and often strive to be perfectionists, regarding poor academic students, although subsequent growth and development in talents are noticed, provide little satisfaction and much self-criticism (Rimm, 2007).

Suicidal thoughts are the least significant symptom among both groups, which is consistent with previous studies (Beard et al., 2016). A meta-analysis study showed that the prevalence of suicidal ideation for Chinese college students is 10.72% (Li et al., 2014). In contrast with the prevalence of depression, 23.8% (Lei et al., 2021), suicidal ideation is found to be less frequent among Chinese college students, which could explain why suicidal thought is the least crucial symptom.

Recent findings are vital to identifying the foremost symptoms of college students with different academic performances. Our study identified that the predominant symptoms in students with poor academic performance were feeling depressed and for students with good academic performance uncontrolled worries. Clinicians who understand the core differences between the two groups could better provide intervention for both groups, psychotherapies such as CBT or Solution Focused Therapy, targeting the key symptom will be most effective (Cramer et al., 2010). The protective factors of emotional well-beings have been mentioned in numerous studies (Haliwa et al., 2022). Coping strategies with positive emotions, mindfulness, and social support are essential protective factors for depression and anxiety (Kim et al., 2021). University administrators and clinicians could focus on enhancing social support, mindfulness and coping strategies for students in order to increase their psychological capital. For lifestyle levels, regular diet, sleep and exercise are also beneficial for psychological well-beings of college students (Lopresti et al., 2013). The healthy lifestyle habits should be encouraged.

The limitations of this study are as noted. First, although the indices of strength had relatively good stability, closeness and betweenness were not stable in both samples, which indicated that we may need to raise the sample size for both samples. Second, our data were collected in a cross-sectional design, the causal relationship and dynamic changes over time cannot be tested, longitudinal design of the study is recommended for future research. Third, although the current study used a stratified cluster sampling method, our study had generalizability problems, our samples were recruited only from three universities in Jiangsu province, China, which may not be able to represent the general population of all Chinese college students.

## 5. Conclusion

In conclusion, our study revealed that network structures are statistically different between students with poor and good academic performance. For the poor academic group, the pertinent symptom is feeling depressed, followed by uncontrolled worry and poor appetite, and for the good academic group, the pertinent symptom is an uncontrolled worry, followed by fatigue or little energy and excessive worry. Theoretical explanations for the group difference have been discussed, and clinical implications have been provided.

## Data availability statement

The original contributions presented in the study are included in the article/Supplementary material, further inquiries can be directed to the corresponding author.

## Ethics statement

The studies involving human participants were reviewed and approved by Yangzhou University. The patients/participants provided their written informed consent to participate in this study.

## Author contributions

YW and XD: study conceptualization. YW: statistical analysis and first drat editing. SZ, XL, and HS: participant recruitment. SZ: writing—review. XD: supervision and project administration. All authors contributed to the article and approved the submitted version.

## Funding

This work is supported by Jiangsu Education Department Distinguished Teacher Project.

## Conflict of interest

The authors declare that the research was conducted in the absence of any commercial or financial relationships that could be construed as a potential conflict of interest.

## Publisher’s note

All claims expressed in this article are solely those of the authors and do not necessarily represent those of their affiliated organizations, or those of the publisher, the editors and the reviewers. Any product that may be evaluated in this article, or claim that may be made by its manufacturer, is not guaranteed or endorsed by the publisher.
