# Supplementary material for: Differences in central symptoms of anxiety and depression between college students with different academic performance: A network analysis
Source: Front Psychol. 2023 Feb 28;14:1071936. doi: 10.3389/fpsyg.2023.1071936 (PMC10011452; doi:10.3389/fpsyg.2023.1071936)

**Supplementary Figure 1. Bootstrapped confidence intervals of edge weights.**


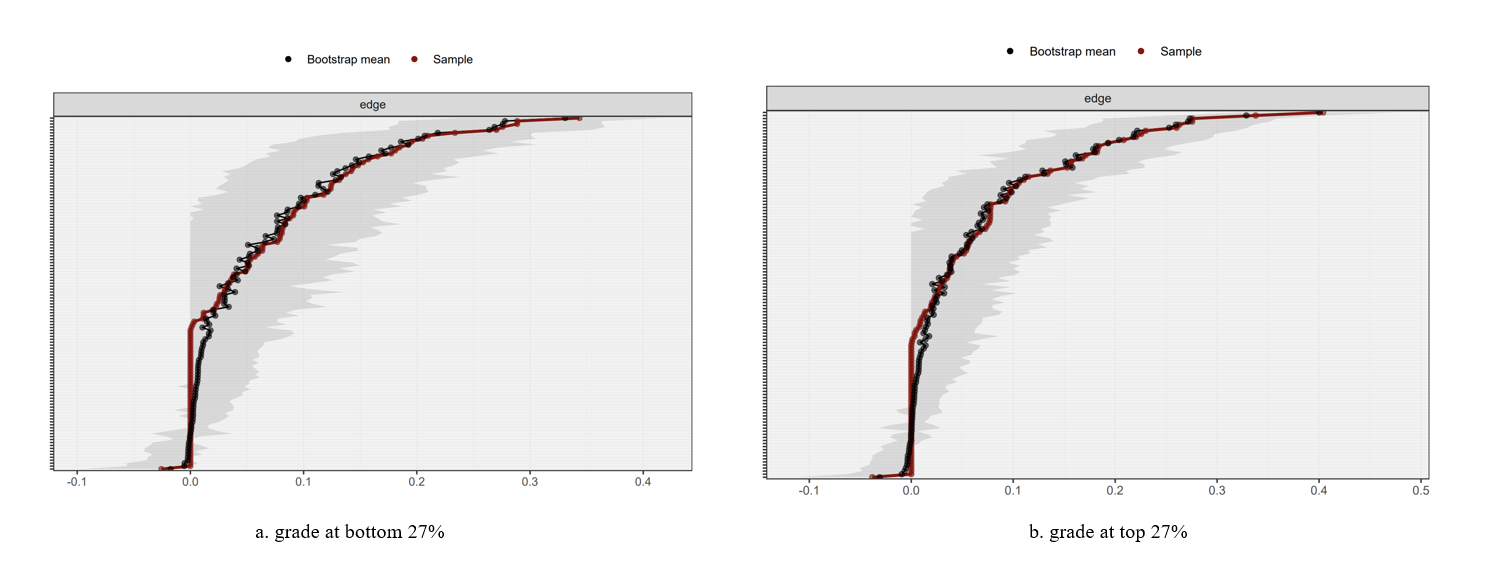


**Supplementary Table 1. Weighted adjacency matrix for poor academic group**

|  | PHQ-1 | PHQ-2 | PHQ-3 | PHQ-4 | PHQ-5 | PHQ-6 | PHQ-7 | PHQ-8 | PHQ-9 | GAD-1 | GAD-2 | GAD-3 | GAD-4 | GAD-5 | GAD-6 | GAD-7 |
| --- | --- | --- | --- | --- | --- | --- | --- | --- | --- | --- | --- | --- | --- | --- | --- | --- |
| PHQ-1 | 0.000 | 0.344 | 0.049 | 0.185 | 0.000 | 0.118 | 0.079 | 0.003 | 0.031 | 0.000 | 0.000 | 0.000 | 0.000 | 0.000 | 0.000 | 0.032 |
| PHQ-2 | 0.344 | 0.000 | 0.057 | 0.148 | 0.000 | 0.131 | 0.026 | 0.000 | 0.088 | 0.079 | 0.000 | 0.093 | 0.101 | 0.000 | 0.000 | 0.082 |
| PHQ-3 | 0.049 | 0.057 | 0.000 | 0.270 | 0.195 | -0.026 | 0.036 | 0.077 | 0.000 | 0.000 | 0.000 | 0.000 | 0.000 | 0.037 | 0.034 | 0.002 |
| PHQ-4 | 0.185 | 0.148 | 0.270 | 0.000 | 0.103 | 0.023 | 0.064 | 0.000 | 0.000 | 0.000 | 0.012 | 0.000 | 0.060 | 0.000 | 0.091 | 0.012 |
| PHQ-5 | 0.000 | 0.000 | 0.195 | 0.103 | 0.000 | 0.124 | 0.153 | 0.091 | 0.081 | 0.000 | 0.000 | 0.000 | 0.000 | 0.026 | 0.021 | 0.000 |
| PHQ-6 | 0.118 | 0.131 | -0.026 | 0.023 | 0.124 | 0.000 | 0.050 | 0.000 | 0.103 | 0.025 | 0.137 | 0.051 | 0.051 | 0.000 | 0.000 | 0.081 |
| PHQ-7 | 0.079 | 0.026 | 0.036 | 0.064 | 0.153 | 0.050 | 0.000 | 0.289 | 0.000 | 0.000 | 0.000 | 0.011 | 0.001 | 0.063 | 0.000 | 0.000 |
| PHQ-8 | 0.003 | 0.000 | 0.077 | 0.000 | 0.091 | 0.000 | 0.289 | 0.000 | 0.142 | 0.000 | 0.000 | 0.000 | 0.000 | 0.234 | 0.000 | 0.053 |
| PHQ-9 | 0.031 | 0.088 | 0.000 | 0.000 | 0.081 | 0.103 | 0.000 | 0.142 | 0.000 | 0.064 | 0.000 | 0.000 | 0.000 | 0.022 | 0.000 | 0.125 |
| GAD-1 | 0.000 | 0.079 | 0.000 | 0.000 | 0.000 | 0.025 | 0.000 | 0.000 | 0.064 | 0.000 | 0.289 | 0.210 | 0.120 | 0.000 | 0.143 | 0.000 |
| GAD-2 | 0.000 | 0.000 | 0.000 | 0.012 | 0.000 | 0.137 | 0.000 | 0.000 | 0.000 | 0.289 | 0.000 | 0.193 | 0.205 | 0.038 | 0.083 | 0.101 |
| GAD-3 | 0.000 | 0.093 | 0.000 | 0.000 | 0.000 | 0.051 | 0.011 | 0.000 | 0.000 | 0.210 | 0.193 | 0.000 | 0.177 | 0.000 | 0.165 | 0.084 |
| GAD-4 | 0.000 | 0.101 | 0.000 | 0.060 | 0.000 | 0.051 | 0.001 | 0.000 | 0.000 | 0.120 | 0.205 | 0.177 | 0.000 | 0.157 | 0.133 | 0.030 |
| GAD-5 | 0.000 | 0.000 | 0.037 | 0.000 | 0.026 | 0.000 | 0.063 | 0.234 | 0.022 | 0.000 | 0.038 | 0.000 | 0.157 | 0.000 | 0.181 | 0.276 |
| GAD-6 | 0.000 | 0.000 | 0.034 | 0.091 | 0.021 | 0.000 | 0.000 | 0.000 | 0.000 | 0.143 | 0.083 | 0.165 | 0.133 | 0.181 | 0.000 | 0.124 |
| GAD-7 | 0.032 | 0.082 | 0.002 | 0.012 | 0.000 | 0.081 | 0.000 | 0.053 | 0.125 | 0.000 | 0.101 | 0.084 | 0.030 | 0.276 | 0.124 | 0.000 |

**Supplementary Table 2. Weighted adjacency matrix for good academic group**

|  | PHQ-1 | PHQ-2 | PHQ-3 | PHQ-4 | PHQ-5 | PHQ-6 | PHQ-7 | PHQ-8 | PHQ-9 | GAD-1 | GAD-2 | GAD-3 | GAD-4 | GAD-5 | GAD-6 | GAD-7 |
| --- | --- | --- | --- | --- | --- | --- | --- | --- | --- | --- | --- | --- | --- | --- | --- | --- |
| PHQ-1 | 0.000 | 0.276 | 0.000 | 0.225 | 0.038 | 0.110 | 0.115 | 0.041 | 0.000 | 0.000 | 0.000 | 0.000 | 0.000 | 0.000 | 0.019 | 0.000 |
| PHQ-2 | 0.276 | 0.000 | 0.036 | 0.156 | 0.092 | 0.183 | 0.000 | 0.077 | 0.038 | 0.020 | 0.078 | 0.008 | 0.077 | 0.000 | 0.005 | 0.000 |
| PHQ-3 | 0.000 | 0.036 | 0.000 | 0.338 | 0.168 | 0.074 | 0.026 | 0.054 | 0.022 | 0.000 | 0.000 | 0.000 | 0.000 | 0.035 | 0.009 | 0.000 |
| PHQ-4 | 0.225 | 0.156 | 0.338 | 0.000 | 0.038 | 0.001 | 0.095 | 0.013 | 0.000 | 0.077 | 0.000 | 0.000 | 0.073 | -0.038 | 0.063 | 0.000 |
| PHQ-5 | 0.038 | 0.092 | 0.168 | 0.038 | 0.000 | 0.059 | 0.136 | 0.057 | 0.032 | 0.000 | 0.000 | 0.001 | 0.000 | 0.000 | 0.020 | 0.000 |
| PHQ-6 | 0.110 | 0.183 | 0.074 | 0.001 | 0.059 | 0.000 | 0.097 | 0.097 | 0.134 | 0.000 | 0.040 | 0.027 | 0.011 | 0.000 | 0.067 | 0.055 |
| PHQ-7 | 0.115 | 0.000 | 0.026 | 0.095 | 0.136 | 0.097 | 0.000 | 0.184 | 0.000 | 0.000 | 0.000 | 0.029 | 0.003 | 0.076 | 0.014 | 0.000 |
| PHQ-8 | 0.041 | 0.077 | 0.054 | 0.013 | 0.057 | 0.097 | 0.184 | 0.000 | 0.171 | 0.000 | 0.000 | 0.000 | 0.000 | 0.220 | 0.000 | 0.000 |
| PHQ-9 | 0.000 | 0.038 | 0.022 | 0.000 | 0.032 | 0.134 | 0.000 | 0.171 | 0.000 | 0.000 | 0.000 | 0.000 | 0.000 | 0.003 | 0.044 | 0.157 |
| GAD-1 | 0.000 | 0.020 | 0.000 | 0.077 | 0.000 | 0.000 | 0.000 | 0.000 | 0.000 | 0.000 | 0.275 | 0.208 | 0.182 | 0.000 | 0.056 | 0.023 |
| GAD-2 | 0.000 | 0.078 | 0.000 | 0.000 | 0.000 | 0.040 | 0.000 | 0.000 | 0.000 | 0.275 | 0.000 | 0.404 | 0.153 | 0.052 | 0.092 | 0.103 |
| GAD-3 | 0.000 | 0.008 | 0.000 | 0.000 | 0.001 | 0.027 | 0.029 | 0.000 | 0.000 | 0.208 | 0.404 | 0.000 | 0.263 | 0.009 | 0.028 | 0.077 |
| GAD-4 | 0.000 | 0.077 | 0.000 | 0.073 | 0.000 | 0.011 | 0.003 | 0.000 | 0.000 | 0.182 | 0.153 | 0.263 | 0.000 | 0.105 | 0.078 | 0.000 |
| GAD-5 | 0.000 | 0.000 | 0.035 | -0.038 | 0.000 | 0.000 | 0.076 | 0.220 | 0.003 | 0.000 | 0.052 | 0.009 | 0.105 | 0.000 | 0.260 | 0.230 |
| GAD-6 | 0.019 | 0.005 | 0.009 | 0.063 | 0.020 | 0.067 | 0.014 | 0.000 | 0.044 | 0.056 | 0.092 | 0.028 | 0.078 | 0.260 | 0.000 | 0.193 |
| GAD-7 | 0.000 | 0.000 | 0.000 | 0.000 | 0.000 | 0.055 | 0.000 | 0.000 | 0.157 | 0.023 | 0.103 | 0.077 | 0.000 | 0.230 | 0.193 | 0.000 |

**Supplementary Table 3. Predictability index for good academic group**

| Scale | Items | Predictability |
| --- | --- | --- |
| PHQ-9 | 1. Little interest or pleasure in doing things? | 64.50% |
|  | 2. Feeling down, depressed, or hopeless? | 57.40% |
|  | 3. Trouble falling or staying asleep, or sleeping too much? | 43.10% |
|  | 4. Feeling tired or having little energy? | 59.90% |
|  | 5. Poor appetite or overeating? | 46.40% |
|  | 6. Feeling bad about yourself — or that you are a failure or have let yourself or your family down? | 39.20% |
|  | 7. Trouble concentrating on things, such as reading the newspaper or watching television? | 60.90% |
|  | 8. Moving or speaking so slowly that other people could have noticed? Or so fidgety or restless that you have been moving a lot more than usual? | 66.50% |
|  | 9. Thoughts that you would be better off dead, or thoughts of hurting yourself in some way? | 85.90% |
| GAD-7 | 1.Feeling nervous, anxious or on edge | 61.00% |
|  | 2.Not being able to stop or control worrying | 54.50% |
|  | 3.Worrying too much about different things | 58.60% |
|  | 4.Trouble relaxing | 52.60% |
|  | 5.Being so restless that it is hard to sit still | 69.00% |
|  | 6.Becoming easily annoyed or irritability | 48.90% |
|  | 7.Feeling afraid as if something awful might happen | 69.70% |

**Supplementary Table 4. Predictability index for poor academic group**

| Scale | Items | Predictability |
| --- | --- | --- |
| PHQ-9 | 1. Little interest or pleasure in doing things? | 70.30% |
|  | 2. Feeling down, depressed, or hopeless? | 57.50% |
|  | 3. Trouble falling or staying asleep, or sleeping too much? | 46.10% |
|  | 4. Feeling tired or having little energy? | 63.80% |
|  | 5. Poor appetite or overeating? | 36.00% |
|  | 6. Feeling bad about yourself — or that you are a failure or have let yourself or your family down? | 46.90% |
|  | 7. Trouble concentrating on things, such as reading the newspaper or watching television? | 58.10% |
|  | 8. Moving or speaking so slowly that other people could have noticed? Or so fidgety or restless that you have been moving a lot more than usual? | 52.20% |
|  | 9. Thoughts that you would be better off dead, or thoughts of hurting yourself in some way? | 69.70% |
| GAD-7 | 1.Feeling nervous, anxious or on edge | 72.40% |
|  | 2.Not being able to stop or control worrying | 66.10% |
|  | 3.Worrying too much about different things | 68.80% |
|  | 4.Trouble relaxing | 64.00% |
|  | 5.Being so restless that it is hard to sit still | 65.50% |
|  | 6.Becoming easily annoyed or irritability | 59.20% |
|  | 7.Feeling afraid as if something awful might happen | 65.10% |

**Supplementary Figure 2.** **Estimation of node strength difference by bootstrapped difference test.**


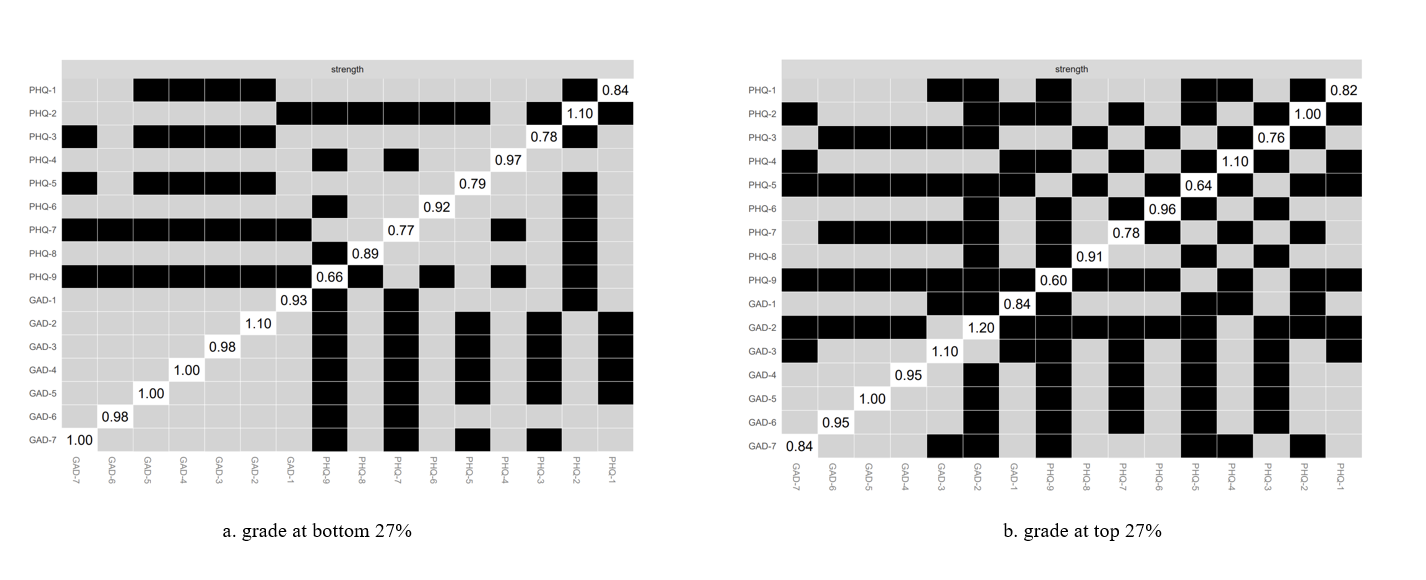

Supplement: Supplementary file 1 [file Data_Sheet_1.docx]
